# Comparative analysis of differentially abundant proteins quantified by LC–MS/MS between flash frozen and laser microdissected OCT-embedded breast tumor samples

**DOI:** 10.1186/s12014-020-09300-y

**Published:** 2020-11-07

**Authors:** Lori A. Sturtz, Guisong Wang, Punit Shah, Richard Searfoss, Praveen-Kumar Raj-Kumar, Jeffrey A. Hooke, J. Leigh Fantacone-Campbell, Brenda Deyarmin, Mary Lou Cutler, Rangaprasad Sarangarajan, Niven R. Narain, Hai Hu, Michael A. Kiebish, Albert J. Kovatich, Craig D. Shriver

**Affiliations:** 1 Chan Soon-Shiong Institute of Molecular Medicine at Windber, Windber, PA USA; 2 Murtha Cancer Center/Research Program, Uniformed Services University of the Health Sciences and Walter Reed National Military Medical Center, Bethesda, MD USA; 3 Department of Surgery, Uniformed Services University of the Health Sciences, Bethesda, MD USA; 4 Henry M. Jackson Foundation for the Advancement of Military Medicine, Bethesda, MD USA; 5 BERG, Framingham, MA USA; 6 Department of Pathology, Uniformed Services University of the Health Sciences, Bethesda, MD USA

**Keywords:** Flash-frozen, Optimal cutting temperature compound, Laser microdissection, Proteomics, Breast tumor, Stroma

## Abstract

**Background:**

Proteomic studies are typically conducted using flash-frozen (FF) samples utilizing tandem mass spectrometry (MS). However, FF specimens are comprised of multiple cell types, making it difficult to ascertain the proteomic profiles of specific cells. Conversely, OCT-embedded (Optimal Cutting Temperature compound) specimens can undergo laser microdissection (LMD) to capture and study specific cell types separately from the cell mixture. In the current study, we compared proteomic data obtained from FF and OCT samples to determine if samples that are stored and processed differently produce comparable results.

**Methods:**

Proteins were extracted from FF and OCT-embedded invasive breast tumors from 5 female patients. FF specimens were lysed via homogenization (FF/HOM) while OCT-embedded specimens underwent LMD to collect only tumor cells (OCT/LMD-T) or both tumor and stromal cells (OCT/LMD-TS) followed by incubation at 37 °C. Proteins were extracted using the illustra triplePrep kit and then trypsin-digested, TMT-labeled, and processed by two-dimensional liquid chromatography-tandem mass spectrometry (2D LC–MS/MS). Proteins were identified and quantified with Proteome Discoverer v1.4 and comparative analyses performed to identify proteins that were significantly differentially expressed amongst the different processing methods.

**Results:**

Among the 4,950 proteins consistently quantified across all samples, 216 and 171 proteins were significantly differentially expressed (adjusted p-value < 0.05; |log_2_ FC|> 1) between FF/HOM vs. OCT/LMD-T and FF/HOM vs. OCT/LMD-TS, respectively, with most proteins being more highly abundant in the FF/HOM samples. PCA and unsupervised hierarchical clustering analysis with these 216 and 171 proteins were able to distinguish FF/HOM from OCT/LMD-T and OCT/LMD-TS samples, respectively. Similar analyses using significantly differentially enriched GO terms also discriminated FF/HOM from OCT/LMD samples. No significantly differentially expressed proteins were detected between the OCT/LMD-T and OCT/LMD-TS samples but trended differences were detected.

**Conclusions:**

The proteomic profiles of the OCT/LMD-TS samples were more similar to those from OCT/LMD-T samples than FF/HOM samples, suggesting a strong influence from the sample processing methods. These results indicate that in LC–MS/MS proteomic studies, FF/HOM samples exhibit different protein expression profiles from OCT/LMD samples and thus, results from these two different methods cannot be directly compared.

## Background

Clinical tissue specimens are a valuable resource for cancer research and can be preserved using multiple types of storage media. For histological analysis and diagnosis, surgical specimens are most often preserved in formalin-fixed paraffin-embedded medium. After clinical evaluation has been completed, specimens with excess material may be preserved and stored for research by flash-freezing (FF) the sample in liquid nitrogen. Alternatively, tissue specimens may be preserved by embedding them in OCT (Optimal Cutting Temperature) compound.

Both FF and OCT-embedded specimens yield high-quality nucleic acids and proteins for downstream molecular analyses. For proteomic analyses, however, FF has been the preferred storage method for protein extraction and processing as OCT can interfere with the performance of liquid chromatography-tandem mass spectrometry (LC–MS/MS) [1]. OCT contains the water-soluble synthetic polymers polyvinyl alcohol (PVA) and polyethylene glycol (PEG) that can compete with peptides during mass spectrometry (MS) analysis to impede chromatographic separation and suppress ion formation, leading to decreased sensitivity and fewer peptide identifications [2–5]. Consequently, several methods to remove OCT have been published that have resulted in an improved number of protein identifications by MS [1–5].

An important component of cancer research is understanding the contribution of various cell types to tumorigenesis. Tissues are comprised of multiple cell types, each of which can possess different molecular profiles at the DNA, RNA, and protein levels. Tumor specimens for research typically contain both the malignant tumor and surrounding “normal” adjacent or stromal tissue. Although not malignant themselves, cells in the surrounding stroma or tumor microenvironment can play a pivotal role in the initiation, promotion, and metastatic potential of the tumor [6–10]. Thus, in order to better understand tumorigenesis and develop appropriate therapies, it is important to study not only the tumor itself, but also the cells in its surrounding microenvironment [8, 11].

FF specimens may provide the best representation of the “tumor as an organ” [12], an important concept to better understand tumorigenesis. In order to fully comprehend the contribution of the different parts of the “organ”, however, we also need to be able to study these components separately from one another. Unfortunately, FF specimens contain a mixture of multiple cell types and are typically lysed using physical disruption methods such as homogenization, sonication, or manual grinding with a mortar and pestle [13], resulting in a homogeneous mixture, making it difficult to ascertain the molecular profiles of specific cell types. On the other hand, although OCT-embedded specimens may also contain a variety of cell types, they can undergo cryosectioning, which not only permits the histological evaluation of the tissue specimens but also allows the samples to undergo laser microdissection (LMD).

LMD techniques have become increasingly popular in cancer research to study the contribution of specific cell types to disease. LMD methods allow for individual cells or cell types to be collected for downstream molecular analyses, including genomics and proteomics [14–16]. Such approaches also preclude the potential of molecular profiles of certain cells being masked by those of others when analyzed as a mixture, such as the tumor and stroma being processed together instead of as separate entities. LMD also allows one to study molecular heterogeneity within the tumor, which along with tumor-stromal interactions, can impact response to treatment [17].

The LMD process is also an effective means of removing OCT from OCT-embedded specimens, thus making these samples compatible with proteomic analysis by MS. An increasing number of proteomic studies are being performed using OCT-embedded tissues following LMD [15, 18–21]. However, with the bulk of proteomic data historically being derived from FF tissues, we asked whether proteomic data derived from FF and OCT samples can be combined and analyzed together in the same study, and therefore, we designed the current study to address this question.

## Methods

### Protein extraction

Five female patients diagnosed with invasive breast cancer were included in this study. Three had tumors of the Luminal A (LA; ER and/or PR + , HER2−, Ki-67 low) subtype while the other two were of the Luminal B1 (LB1; ER and/or PR + , HER2−, Ki-67 high) subtype [22, 23]. For each patient, flash-frozen and OCT-embedded tumor specimens were selected. Flash-frozen samples were homogenized (FF/HOM) with a rotor–stator homogenizer for 30 s or 1 min (2X for 30 s) in 350 µl of Lysis buffer type 15 (from the illustra triplePrep Kit; GE Healthcare Life Sciences) containing 1% β-mercaptoethanol and placed on ice. Homogenized samples were then incubated at room temperature for 5 min to allow any foam to dissipate. Samples were then processed with the illustra triplePrep Kit, which allows for simultaneous extraction of DNA, RNA, and protein. Homogenized lysates were added to the DNA column and the protocol followed according to the manufacturer’s specifications.

For OCT-embedded specimens, tissue sections were cut at 8 µm thickness in a cryostat at −30 °C and placed on PEN membrane slides (Leica Microsystems). Tissue sections were stained using the Histogene™ LCM Frozen Section Staining Kit (Thermo Fisher Scientific). Stained sections then underwent LMD using the Leica LMD7 Laser Microdissection System (Leica Microsystems). For samples designated as “Tumor” (OCT/LMD-T), only tumor cells were microdissected and collected for analysis whereas for “Tumor + Stroma” (OCT/LMD-TS) samples, both the tumor and the surrounding adjacent stromal tissue were microdissected and collected into the same tube. The % tumor and % stroma of each of the five OCT-embedded breast specimens is shown in Additional file 1: Table S1 both immediately prior to LMD (Pre-LMD) and immediately after LMD (Post-LMD), as the tumor and stromal composition of the OCT-embedded specimens may change as serial sections for LMD are cut. The OCT/LMD-T samples contain nearly 100% tumor with little to no stroma whereas the tumor and stromal composition of each of the OCT/LMD-TS samples are represented by the percentages reported in Additional file 1: Table S1. The microdissected samples were collected in 350 µl of Lysis buffer type 15 containing 1% β-mercaptoethanol and incubated for 10 min at 37 °C in a hybridization oven. Following this incubation, the samples were vortexed briefly, spun down, and further processed with the illustra triplePrep Kit, as was done for the flash-frozen specimens.

For all specimen types, the protein pellets were re-suspended in 8 M urea in 100 mM ammonium bicarbonate, pH 7.8. Protein concentrations were measured using the Bradford assay.

### Trypsin digestion

Proteins were reduced with 10 mM Tris(2-carboxyethyl)phosphine (TCEP) for 60 min at 55 °C and alkylated with 18.75 mM iodoacetamide for 30 min at room temperature in the dark. Proteins were then precipitated overnight in acetone, and pellets were reconstituted in 200 mM triethylammonium bicarbonate (TEAB) at 1 mg/mL and digested with trypsin at 1:40 (trypsin:protein) overnight at 37 °C.

### TMT labeling of peptides

**Fig. 1:**
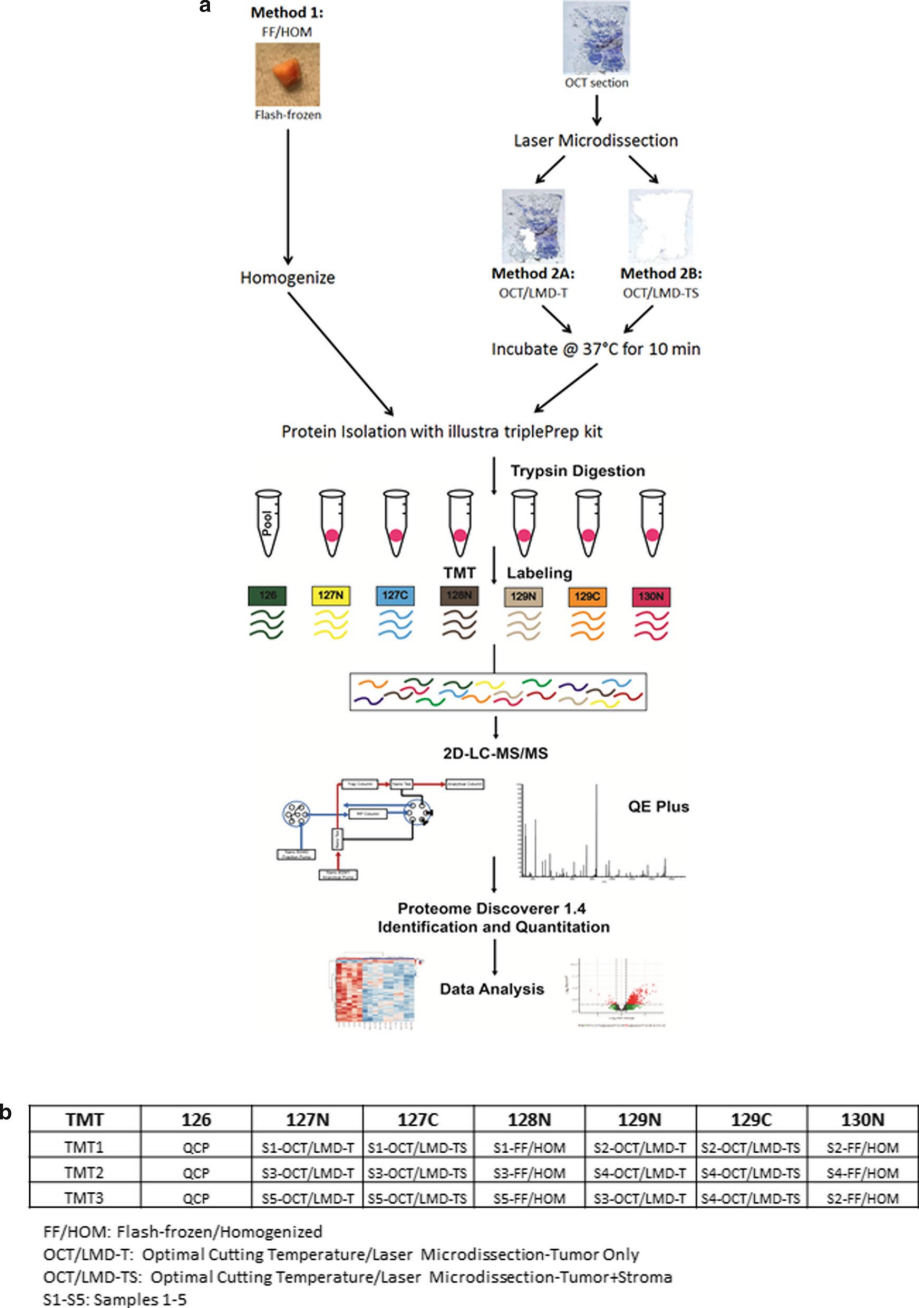
Sample processing workflow. **a** Proteins were extracted from five invasive breast cancer specimens using three different methods, generating a total of 15 protein samples. In Method 1, flash-frozen specimens were homogenized (FF/HOM) and protein isolated using the illustra triplePrep kit. In Methods 2A and 2B, sections from OCT-embedded specimens were laser microdissected to isolate tumor cells only (OCT/LMD-T) or both tumor and stromal cells (OCT/LMD-TS), respectively. In both Methods 2A and 2B, the laser microdissected material was then incubated at 37 °C for 10 min followed by protein isolation using the illustra triplePrep kit. Extracted proteins were trypsin digested and TMT-labeled. TMT-labeled peptides were analyzed using 2D-LC–MS/MS and proteins quantified using Proteome Discoverer v1.4 followed by data analysis to identify differentially expressed proteins among the three different sample storage/processing methods. **b** Sample-to-TMT channel mapping. Samples were analyzed in 3 TMT sets with 7 channels in each set. In each TMT set, channel 126 contained the QCP (quality control pooled sample) while the remaining 6 channels contained the individual TMT-labeled samples. The last three TMT channels in set 3 contained technical replicates, one for each sample storage/preparation method

Digested peptides were labeled with 10-plex TMT (Tandem Mass Tag) reagents (Thermo Fisher Scientific). Peptides (20 µg) suspended in 200 mM TEAB, pH 8.0, at a concentration of 1 mg/mL were mixed with 20 µl of TMT reagent freshly dissolved in anhydrous acetonitrile to a concentration of 20 mg/mL. After a 1-h incubation at room temperature, samples were placed in the refrigerator overnight. A reference sample was created by pooling an aliquot of peptides from each individual FF/HOM, OCT/LMD-T and OCT/LMD-TS sample, and Channel 126 was used for labeling the pooled reference sample throughout the sample analysis. The sample-to-TMT channel mapping is shown in Fig. 1b. The TMT-labeled peptides were then mixed, dried, and desalted on C18 spin columns. Peptides were dried in a vacuum centrifuge and stored at −20 °C until LC–MS/MS analysis.

### Mass spectrometry

LC–MS/MS analysis was performed using a Waters nanoAcquity 2D LC system coupled to a Thermo Q Exactive Plus MS. 5 µg of TMT-labeled peptides were fractionated using two-dimensional reversed-phase liquid chromatography. In the first dimension, 20 mM ammonium formate (Buffer A) and 100% acetonitrile (Buffer B) were used to generate nine fractions by eluting in 16, 20, 24, 26, 28, 30, 32, 36, and 50% of Buffer B, respectively. In the second dimension, fractions were separated over a 145-min gradient with a change of 20–23%, using 0.1% formic acid in water as Buffer A and 0.1% formic acid in acetonitrile as Buffer B. The column eluate was directly introduced into the mass spectrometer via a nano-ESI source, and candidate ions were selected and fragmented using a data-dependent Top-15 acquisition method. Full MS survey scans were performed at a resolution of 70,000 with a scan range of 400–1800 Thomsons (Th; Th = Da/z). MS/MS scans were collected at a resolution of 35,000 with a 1.2 Th isolation window. In order for an ion to be considered a candidate for fragmentation, it had to be assigned a charge in the range of + 2 to + 4.

### Protein quantification

Raw LC–MS/MS data were processed using Proteome Discoverer v1.4 (Thermo Scientific) and searched against the RefSeq protein database using the database search algorithm SEQUEST to identify and quantify proteins. Only unique peptides were used for protein quantification, and two such peptides were required to identify a protein. The search parameters included: (1) tryptic peptides of ≥ 6 amino acids in length and up to two missed cleavage sites, (2) precursor mass tolerance of 10 ppm, (3) fragment mass tolerance of 0.02 Da, (4) static modifications including cysteine carbamidomethylation and N-terminal TMT-10 plex, and (5) dynamic modifications including asparagine and glutamine deamidation, methionine oxidation, and lysine TMT-10 plex.

### Sample quality control and normalization

Protein-level abundances in terms of TMT ratios were log_2_-transformed, and the sample quality control (QC) of quantified proteins was performed using density plot and dip statistics, which demonstrated that all samples conformed to an expected unimodal Gaussian distribution. Samples were normalized using a 2-component Gaussian mixture model-based method [24]. Briefly, the normalization procedure centers the distribution of log_2_-transformed ratios on zero to nullify the effect of differential protein loading or systematic MS variation. The normalized protein abundances of technical replicates were merged at the sample level using median values. A total of 4,950 proteins expressed in all 15 samples were extracted for further analysis. Principal component analyses (PCA) were performed with significant proteins as indicated. For hierarchical clustering, Euclidian distance was used as the distance matrix and Ward was used as the linkage method.

### Differential analysis of FF/HOM versus OCT/LMD samples

The moderated t-test, implemented in limma [25] Bioconductor package [26] (version 3.38.3), was used for subtype-adjusted comparative analyses (FF/HOM versus OCT/LMD-T samples; FF/HOM versus OCT/LMD-TS samples). The significantly differentially expressed proteins for FF/HOM samples versus OCT/LMD-T samples were identified with a Benjamini-Hochberg (BH) adjusted p-value < 0.05 and absolute log_2_ fold change (|log_2_ FC|) > 1. The same analysis procedure was applied to identify significantly altered proteins between FF/HOM samples and OCT/LMD-TS samples. The significantly differentially expressed proteins in both FF/HOM versus OCT/LMD-T and FF/HOM versus OCT/LMD-TS samples were utilized as a signature to distinguish OCT/LMD samples from FF/HOM samples and for PCA and clustering analyses.

### Differential analysis of OCT/LMD-T versus OCT/LMD-TS samples

In addition to using subtype-adjusted analysis similar to that for FF/HOM versus OCT/LMD, the more sensitive paired analysis was also performed to compare OCT/LMD-T to OCT/LMD-TS samples. Because no statistically significant differentially expressed proteins were detected at an adjusted p-value < 0.05 and |log_2_ FC|> 1, an adjusted p-value < 0.4 and |log_2_ FC|> 1 were used in the paired analysis to identify trended differentially expressed proteins between OCT/LMD-T and OCT/LMD-TS specimens.

### Single Sample Gene Set Enrichment Analysis (ssGSEA)

Protein accessions were converted to Entrez identifiers using the Gene ID Conversion Tool in DAVID [27]. Gene sets from the MSigDB [28] database v6.2 C5 collection [29] were downloaded on July 11, 2019 for analysis. The MSigDB C5 Collection was derived from the Gene Ontology (GO) annotations, and GO terms with very narrow and very broad categories were filtered out. Only the largest GO term gene set among “highly similar” (Jaccard similarity coefficient > 0.85) GO term gene sets was retained. We further filtered the MSigDB C5 Collection by keeping only GO term gene sets containing at least 5 genes and no more than 1000 genes among the 4,950 proteins. Subsequently, 4,743 GO terms were used for analysis. To identify biologically interpretable features associated with the sample storage/preparation methods, ssGSEA implemented in GSEA [30] Bioconductor package was used to transform the protein abundance matrix to a GO-term enrichment score matrix. The differential analysis with limma [25] was performed on the GO-term enrichment score matrix to identify significantly altered GO terms in each comparison (FF/HOM versus OCT/LMD-T; FF/HOM versus OCT/LMD-TS; and trended differences in OCT/LMD-T versus OCT/LMD-TS), similar to the way we performed the analysis at the protein level. The significance of the GO term gene set differences was determined using an adjusted p-value (BH) < 0.2.

## Results

### Number of detected proteins across samples

To evaluate the impact of sample storage and preparation methods for breast tumor tissues on quantitative proteomic analysis, we compared proteins quantified from five breast tumor specimens processed by two methods in three different ways: FF/HOM, OCT/LMD-T and OCT/LMD-TS (Fig. 1). A total of 7,371 proteins were quantified across the tumor specimens. The number of proteins detected in each sample is shown in Additional file 1: Table S2. Using repeated measures ANOVA, there was no difference in the number of distinct proteins detected across the 15 samples regardless of the storage/processing method that was used (p = 0.174). However, the number of detected proteins did vary significantly across the three TMT experiments (p = 1.51 × 10^–05^). To avoid biases of undetected proteins, we focused our remaining analyses on 4,950 proteins consistently quantified across all tumor samples.

### Differentially expressed proteins between FF/HOM and OCT/LMD samples

**Fig. 2:**
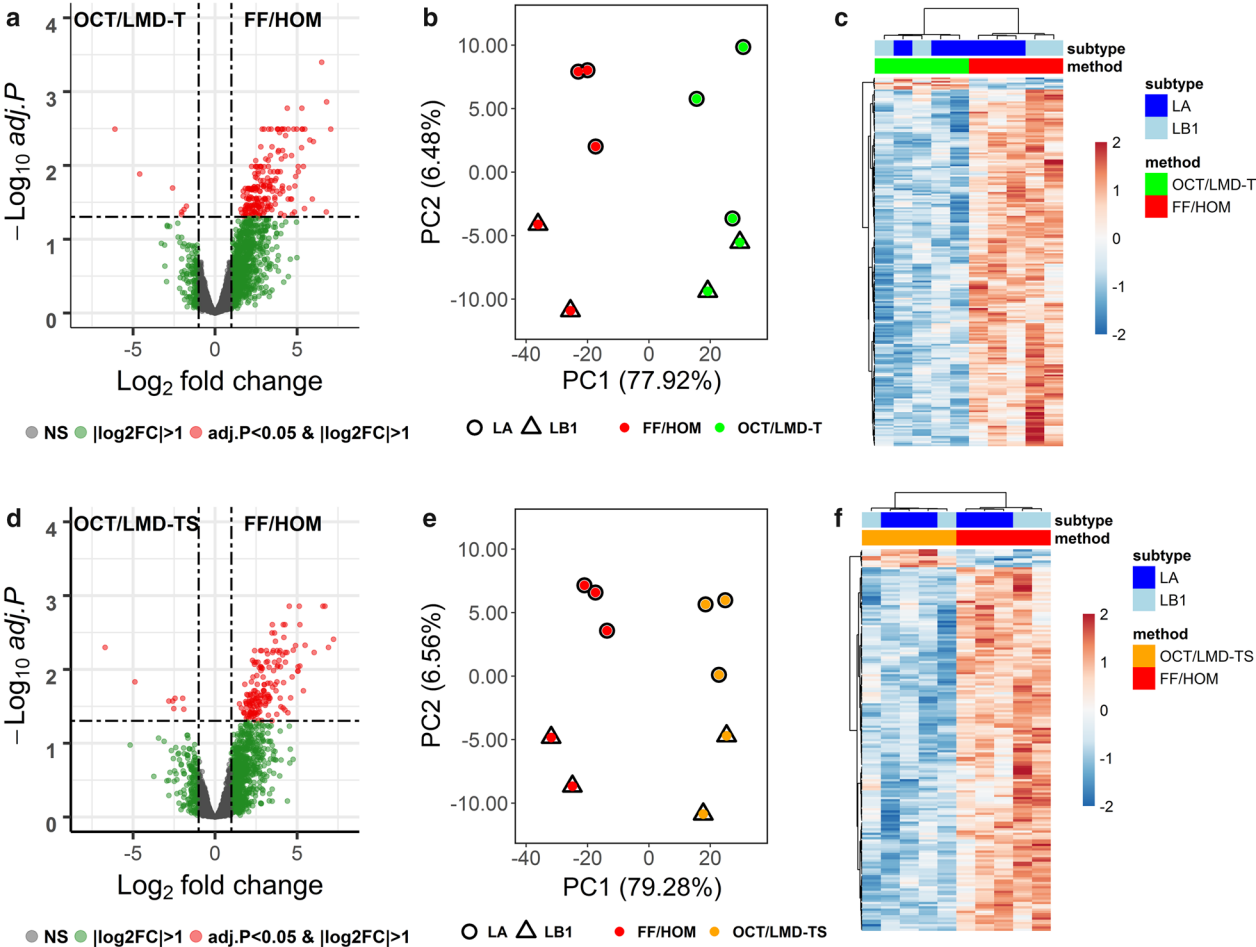
Differentially expressed proteins between FF/HOM and OCT/LMD samples. **a**–**c** FF/HOM vs. OCT/LMD-T and **d**–**f**, FF/HOM vs. OCT/LMD-TS. **a**, **d** Volcano plots showing the distribution of fold changes of protein abundance and adjusted p-value (BH) for each comparison. NS, not significant, here indicating adj. p-value ≥ 0.05 and |log_2_ FC|≤ 1. **b**, **e** PCA analysis of 216 and 171 significant proteins, respectively. **c**, **f** Heat maps generated using unsupervised clustering analysis with 216 and 171 significant proteins, respectively. Each column represents one sample, each row represents a significant protein. LA, luminal A; LB1, luminal B1

Although the number of proteins detected was similar across the samples irrespective of processing method, the expression levels of the quantified proteins were highest in the FF/HOM preparations compared to both the OCT/LMD-T and OCT/LMD-TS samples (Additional file 1: Tables S3 and S4). The comparison between FF/HOM and OCT/LMD-T samples yielded 216 significantly differentially expressed proteins (adjusted p-value (BH) < 0.05 and |log_2_ FC|> 1) (Fig. 2a) with 209 proteins (97%) up-regulated and only 7 proteins (3%) down-regulated in FF/HOM samples (Additional file 1: Table S3). PCA analysis (Fig. 2b) and unsupervised clustering analysis (Fig. 2c) with these 216 proteins demonstrated two distinct clusters separated by storage/preparation methods. Similarly, in the FF/HOM and OCT/LMD-TS comparison, 171 proteins were significantly differentially expressed (adjusted p-value (BH) < 0.05; |log_2_ FC|> 1) (Fig. 2d). Of these 171 proteins, 163 (95%) were up-regulated and only 8 (5%) were down-regulated in FF/HOM (Additional file 1: Table S4). PCA analysis (Fig. 2e) and unsupervised clustering analysis (Fig. 2f) with these 171 proteins showed that these proteins can distinguish OCT/LMD-TS samples from FF/HOM samples.

### Differentially enriched GO terms between FF/HOM and OCT/LMD samples

**Fig. 3:**
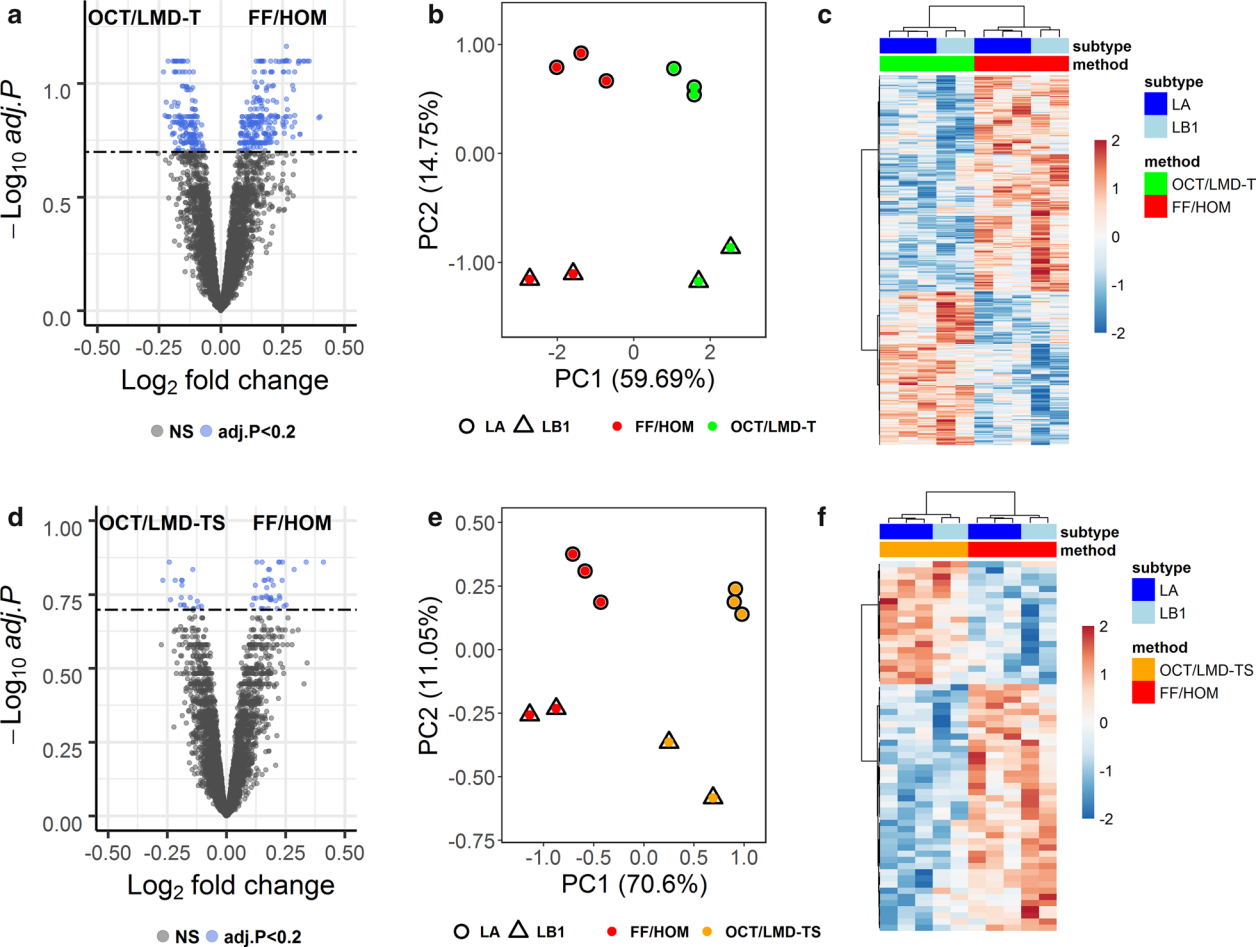
Differentially enriched GO terms between FF/HOM and OCT/LMD samples. **a**–**c** FF/HOM vs. OCT/LMD-T and **d**–**f** FF/HOM vs. OCT/LMD-TS. **a**, **d** Volcano plots showing the distribution of fold changes of GO term abundance and adjusted p-value for each comparison. NS, not significant, here denoting adj. p-value ≥ 0.2. **b**, **e** PCA analysis of 402 and 60 significant GO terms, respectively. **c**, **f** Heat maps generated using unsupervised clustering analysis with 402 and 60 significant GO terms, respectively. Each column represents one sample, each row represents a significant GO term. LA, luminal A; LB1, luminal B1

Out of 4,878 unique genes mapped to 4,950 proteins, 402 GO terms were significantly (adjusted p-value (BH) < 0.2) differentially enriched between FF/HOM and OCT/LMD-T samples (Fig. 3a and Additional file 1: Table S6). In the FF/HOM and OCT/LMD-TS comparison, there were 60 significantly differentially enriched GO terms (adjusted p-value (BH) < 0.2) (Fig. 3d and Additional file 1: Table S7). PCA analysis (Fig. 3b, e) and unsupervised clustering analysis (Fig. 3c, f) using significantly differentially enriched GO terms in each comparison not only distinguished OCT/LMD samples from FF/HOM samples but also separated LA and LB1 breast cancer subtypes within each storage/preparation method from one another.

### Analysis of common proteins and GO terms

**Fig. 4:**
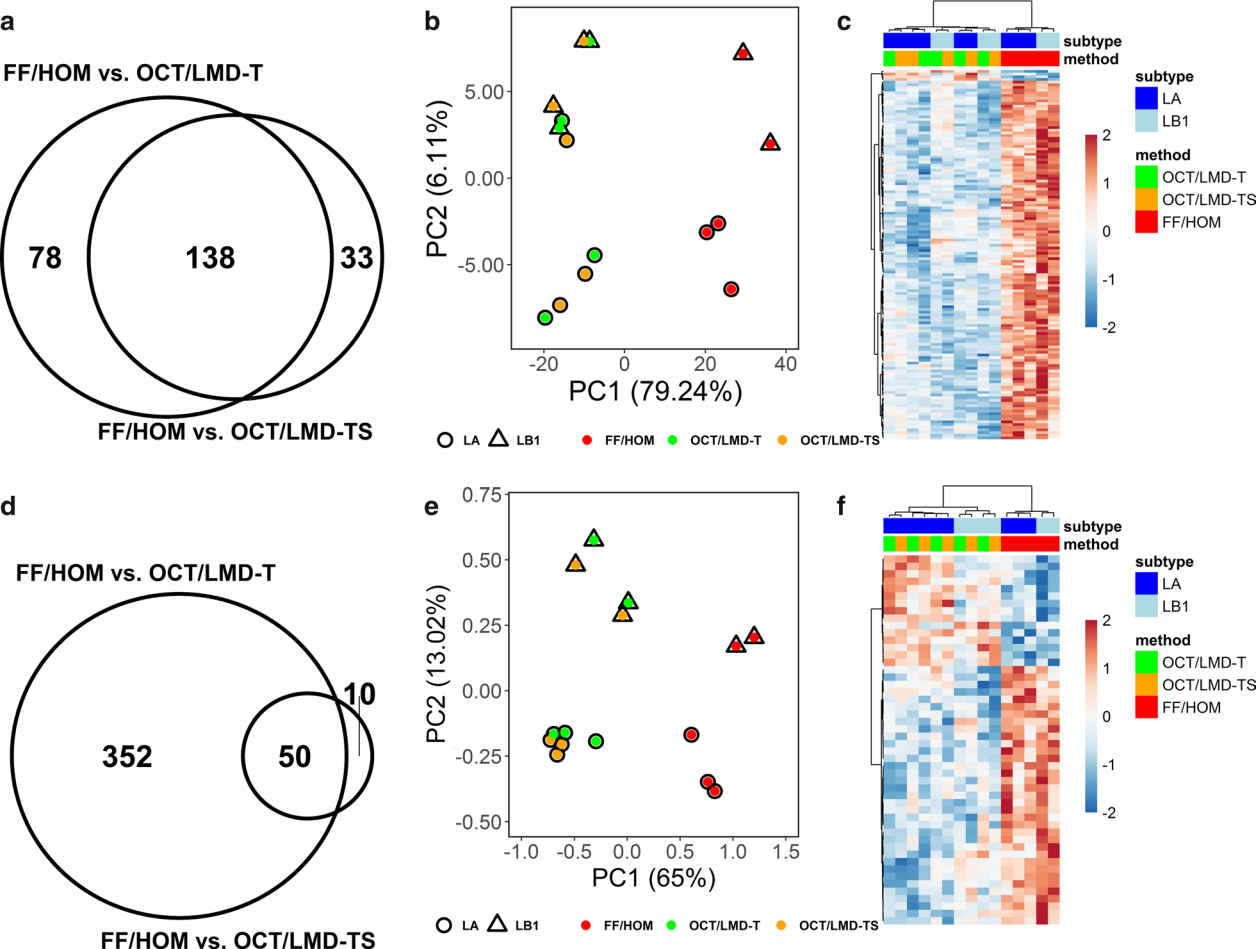
Analysis of common proteins and GO terms between FF/HOM and OCT/LMD comparisons. **a**–**c** Common proteins and **d**–**f** common GO terms. **a** Venn diagram showing the overlap of 216 and 171 significant proteins differentially expressed in FF/HOM vs. OCT/LMD-T and FF/HOM vs. OCT/LMD-TS, respectively. Only proteins with adj. p-value < 0.05 and |log_2_ FC|> 1 were considered significant. **b** PCA analysis using 138 common significant proteins from **a**. **c** Heat map using unsupervised clustering analysis with 138 common significant proteins identified in **a**. Each column represents one sample, each row represents a common significant protein. **d** Venn diagram showing the overlap of 402 and 60 significant GO terms differentially expressed in FF/HOM vs. OCT/LMD-T and FF/HOM vs. OCT/LMD-TS, respectively. Only GO terms with adj. p-value < 0.2 were considered significant. **e** PCA analysis using 50 common significant GO terms from **d**. **f** Heat map using unsupervised clustering analysis with 50 common significant GO terms identified in **d**. Each column represents one sample, each row represents a common significant GO term. LA, luminal A; LB1, luminal B1

Significantly differentially expressed proteins and enriched GO terms common between the FF/HOM and OCT/LMD comparisons were investigated. In Fig. 4a, the Venn diagram shows the overlap between significantly differentially expressed proteins in the FF/HOM vs. OCT/LMD-T and FF/HOM vs. OCT/LMD-TS comparisons. Only proteins with an adjusted p-value (BH) < 0.05 and an |log_2_ FC|> 1 were considered significant. PCA analysis (Fig. 4b) and unsupervised clustering analysis (Fig. 4c) of the 138 common significant proteins (Additional file 1: Table S5) indicates that FF/HOM samples are well-separated from OCT/LMD samples. Similarly, in Fig. 4d, the Venn diagram shows the overlap between significant GO terms differentially enriched in the FF/HOM vs. OCT/LMD-T and FF/HOM vs. OCT/LMD-TS comparisons. Only GO terms with an adjusted p-value (BH) < 0.2 were considered significant. PCA analysis (Fig. 4e) and unsupervised clustering analysis (Fig. 4f) using the 50 common significant GO terms (Additional file 1: Table S8) also indicate that the profiles from FF/HOM samples are distinct from those of OCT/LMD samples. In addition, these analyses were also able to discriminate LA from LB1 subtypes within each storage/preparation method (Fig. 4e, f).

As shown in Fig. 4a, 216 proteins were differentially expressed between FF/HOM and OCT/LMD-T samples whereas only 171 proteins were differentially expressed between FF/HOM and OCT/LMD-TS samples; this difference (216 vs. 171 out of a total of 4,950 proteins) was statistically significant (p = 0.022 using Fisher’s exact test). The difference is even more apparent when looking at the number of differentially enriched GO terms between the comparisons (Fig. 4d). Over 400 GO terms were differentially enriched between FF/HOM and OCT/LMD-T samples whereas only 60 GO terms were different between FF/HOM and OCT/LMD-TS specimens; this difference in the number of significantly differentially enriched GO terms (402 vs. 60 out of 4,743 GO terms) was statistically significant (p < 2.2 × 10^–16^ using Fisher’s exact test). However, although FF/HOM appears to be more similar to OCT/LMD-TS than to OCT/LMD-T based on the number of significantly differentially expressed proteins and enriched GO terms, FF/HOM shows no greater similarity to OCT/LMD-TS than OCT/LMD-T based on PCA analysis with either proteins or GO terms (Fig. 4b, e, based on weighted distance for pairwise samples, p = 0.97 from paired t-test).

### Non-significant differentially expressed proteins between OCT/LMD-T and OCT/LMD-TS samples

We did not identify any significantly differentially expressed proteins between OCT/LMD-T and OCT/LMD-TS samples based on adjusted p-values, suggesting that these sample types are very similar to one another. When using a non-adjusted p-value < 0.05 and an |log_2_ FC|> 1, 65 proteins met these criteria (Additional file 1: Table S9). Of these, 60/65 proteins (92%) were more highly expressed in the OCT/LMD-TS samples. PCA analysis and unsupervised clustering analysis using these 65 proteins demonstrated that OCT/LMD-T samples are not easily distinguishable from OCT/LMD-TS samples (data not shown).

### Non-significant differentially enriched GO terms between OCT/LMD-T and OCT/LMD-TS samples

Similarly, no GO terms were differentially enriched between OCT/LMD-T and OCT/LMD-TS based on adjusted p-values. When using a non-adjusted p-value < 0.05, 213 GO terms were identified (Additional file 1: Table S10), again showing the high similarity between these two sample types. PCA analysis and unsupervised clustering analysis using these 213 GO terms demonstrated patterns similar to those observed with the protein (data not shown).

### Trended differentially expressed proteins and differentially enriched GO terms between OCT/LMD-T and OCT/LMD-TS samples using paired analyses

Using the more conservative subtype-adjusted analysis, we were unable to identify any statistically significantly differentially expressed proteins between OCT/LMD-T and OCT/LMD-TS specimens, which was likely due to a lack of statistical power and thus, a larger sample size may be needed to detect any significant differences within this comparison. Nonetheless, we also compared the OCT/LMD-T and OCT/LMD-TS samples using the more sensitive paired analysis. We identified 66 proteins that trended to be differentially expressed (adjusted p-value (BH) < 0.4 and |log_2_ FC|> 1) (Additional file 1: Table S11). Among these proteins, 62/66 (94%) were more highly expressed in the OCT/LMD-TS samples. Likewise, we found 456 GO terms that trended to be differentially enriched between OCT/LMD-T and OCT/LMD-TS specimens (adjusted p-value (BH) < 0.2) with 339/456 (74%) GO terms being more enriched in the OCT/LMD-TS samples (Additional file 1: Table S12).

We were interested to see what biological processes and functions trended to be differentially enriched between OCT/LMD-T and OCT/LMD-TS samples. As may be expected, among the 456 GO terms were multiple GO terms associated with the stroma or tumor microenvironment [6–11, 31–33], all of which were more highly enriched in the OCT/LMD-TS samples. A subset of these stroma-associated GO terms is shown in Additional file 1: Table S13 along with the proteins from Additional file 1: Table S11 that map to them.

## Discussion

In the present study, we compared proteomic profiles obtained from two standard storage and processing methods [1, 13–16, 34] for frozen tissue specimens: (1) FF samples lysed via homogenization and (2) laser microdissected OCT-embedded specimens lysed via incubation at 37 °C, followed by protein extraction from both using the same isolation kit. Our goal was to determine if MS data from these two different sample types can be combined and analyzed together in the same study. We had expected to find that the OCT/LMD-TS samples were more similar to the FF/HOM samples, but in fact, found that they were more similar to the OCT/LMD-T specimens, most likely due to differences in the sample processing methods of the two specimen types. Thus, proteomic data obtained from frozen specimens stored and processed differently as described here cannot be integrated and analyzed in the same study.

Surprisingly, as measured by the differentially expressed proteins and differentially enriched GO terms, the OCT/LMD-T and OCT/LMD-TS samples were more similar to each other than either was to the FF/HOM samples. The % tumor nuclei in the OCT/LMD samples ranged from 50–75%. Based on this information, we would expect the stromal content of the OCT/LMD-TS samples to range from approximately 25–50%. Presuming that the tumor and stromal content of the FF/HOM and OCT/LMD-TS samples are very comparable to one another, we predicted that the OCT/LMD-TS samples would have shown greater similarities to the FF/HOM samples in protein expression than what was observed in this study. This unexpected observation is likely due to the methods used to process the samples (homogenization vs. LMD/incubation).

We observed that among the significantly differentially expressed proteins that most were more abundant in the FF/HOM samples compared to both the OCT/LMD-T and OCT/LMD-TS samples. We were curious about the relative abundance of the non-significant proteins among the 4,950 consistently-detected proteins and found that the majority of these proteins were also more abundant in the FF/HOM samples in all five tumors that were studied. A representative example is shown in Additional file 2: Figure S1. It is possible that protein recovery in the FF/HOM specimens is greater due to homogenization being a more effective method of lysing the sample [13] and releasing proteins for extraction from the cells and/or intracellular organelles. Although these results strongly suggest that some difference in the processing methods is responsible for the increased abundance of certain proteins observed in FF/HOM vs. OCT/LMD samples, we are not yet able to pinpoint the specific causative step(s) until new, specifically designed experiments are conducted. For example, we may compare FF/HOM to whole sections of OCT/TS processed by homogenization or incubation at 37 °C which will provide insight as to whether the storage method or the processing method is responsible for the observed difference in protein abundance. However, this experiment is beyond the scope of the current study.

The primary goal of the current study was to determine if proteomic data obtained from FF/HOM and OCT/LMD specimens could be integrated and analyzed together in the same study. Based on previous unpublished experiments comparing FF/HOM and OCT/LMD-T of unpaired samples at batches of n = 5, we determined that 5 samples were sufficient to identify differences between FF/HOM and OCT/LMD-T in paired samples, and in the current study, significantly differentially expressed proteins and enriched GO terms between FF/HOM and OCT/LMD specimens were indeed detected. However, this design was not powered to discriminate OCT/LMD-T from OCT/LMD-TS samples. Using the more sensitive paired analysis, however, we were able to detect trends in differentially expressed proteins and enriched GO terms at adjusted p-value < 0.4, suggesting that with a larger sample size we may obtain the statistical power needed to identify significantly differentially expressed proteins between OCT/LMD-T and OCT/LMD-TS samples. Moreover, although our results suggested that FF/HOM specimens were more similar to OCT/LMD-TS than OCT/LMD-T specimens based on the number of differentially expressed proteins and enriched GO terms, the PCA analysis did not show that there was greater similarity between FF/HOM and OCT/LMD-TS samples. Our inability to make a firm conclusion as to whether FF/HOM samples are indeed more similar to OCT/LMD-TS than OCT/LMD-T samples is not surprising as the current study was not designed for this purpose.

Finally, when designing the current study, we included three breast tumors of the LA subtype and two of the LB1 subtype among the five breast cancer cases. Patients with LA tumors tend to have the best prognosis with high survival rates and low recurrence rates [35–38], whereas patients with LB1 tumors tend to have a poorer prognosis [35–38]. Even with no statistical power in this small sample set, both the PCA analysis and the unsupervised clustering analysis were able to separate LA from LB1 tumors, particularly in the FF/HOM samples and in all samples when using GO terms for the analysis. These results support pursuing future, larger proteomic studies to compare the LA and LB1 subtypes, and other breast cancer subtypes as well.

## Conclusions

We investigated whether proteomic data derived from two standard types of frozen tissue specimens could be combined and analyzed together in the same study. We found that proteomic profiles of the FF/HOM specimens are distinct from those of the OCT/LMD specimens, and thus, data from these two types of samples cannot be integrated for analysis. These results provide guidance for designing new experiments to explore whether the more abundant protein expression observed in FF/HOM samples compared to OCT/LMD samples is due to sample storage (FF vs. OCT) or sample processing (HOM vs. LMD), which may advance our understanding as to whether a method could eventually be developed to integrate proteomic data using these two standard tumor tissue storage and processing methods. Our results also provide preliminary data to support the design of new experiments to address important cancer biology questions including the exploration of the differences between OCT/LMD-T and OCT/LMD-TS samples as well as between LA and LB1 subtypes of breast tumors.

## Supplementary information

**Additional file 1: Figure S1.** Scatter plots of normalized protein expression values of sample S2 in FF/HOM vs. OCT/LMD-TS processing methods. Each dot represents one protein. Numbers in red represent the number of more abundant proteins in FF/HOM whereas numbers in blue represent the number of more abundant proteins in OCT/LMD-TS. A, Scatter plot of the reported significant proteins. B, Scatter plot of the non-significant proteins from the 4,950 proteins detected across all samples. C, Scatter plot of the proteins detected in both methods but not in the 4,950 commonly detected proteins. D, Scatter plot of the proteins detected in only one of the methods.

**Additional file 2: Table S1.** Percentage of tumor and stroma in each OCT/LMD sample before and after laser microdissection. **Table S2.** Number of proteins quantified in each sample. **Table S3.** Differentially expressed proteins between FF/HOM and OCT/LMD-T. **Table S4.** Differentially expressed proteins between FF/HOM and OCT/LMD-TS. **Table S5.** Differentially expressed proteins common between FF/HOM vs OCT/LMD-T and FF/HOM vs. OCT/LMD-TS. **Table S6.** Differentially enriched GO Terms between FF/HOM and OCT/LMD-T. **Table S7.** Differentially enriched GO Terms between FF/HOM and OCT/LMD-TS. **Table S8.** Differentially enriched GO Terms common between FF/HOM vs OCT/LMD-T and FF/HOM vs. OCT/LMD-TS. **Table S9.** Non-significant differentially expressed proteins between OCT/LMD-T and OCT/LMD-TS. **Table S10.** Non-significant differentially enriched GO Terms between OCT/LMD-T and OCT/LMD-TS. **Table S11.** Trended differentially expressed proteins between OCT/LMD-T and OCT/LMD-TS using paired analysis. **Table S12.** Differentially enriched GO Terms between OCT/LMD-T and OCT/LMD-TS using paired analysis. **Table S13.** Select stroma-associated GO terms from Table S12 and the proteins from Table S11 that map to them.

## Data Availability

The datasets used and/or analyzed during the current study are available from the corresponding author upon reasonable request.

## Abbreviations

2D LC–MS/MS: Two-dimensional liquid chromatography-tandem mass spectrometry
BH: Benjamini-Hochberg
FC: Fold-change
FF: Flash-frozen
GO: Gene ontology
HOM: Homogenized
LA: Luminal A breast cancer subtype
LB1: Luminal B1 breast cancer subtype
LMD: Laser microdissection
LMD-T: Laser microdissection of tumor only
LMD-TS: Laser microdissection of both tumor and surrounding stroma
MS: Mass spectrometry
OCT: Optimal cutting temperature compound
PCA: Principal component analysis
PEG: Polyethylene glycol
PVA: Polyvinyl alcohol
ssGSEA: Single sample gene set enrichment analysis
TCEP: Tris(2-carboxyethyl)phosphine
TEAB: Triethylammonium bicarbonate
Th: Thomsons
TMT: Tandem mass tag
QC: Quality control

## References

[1] Vrana M, Goodling A, Afkarian M, Prasad B (2016). An optimized method for protein extraction from OCT-embedded human kidney tissue for protein quantification by LC-MS/MS proteomics. Drug Metab Dispos..

[2] Shah P, Zhang B, Choi C, Yang S, Zhou J, Harlan R (2015). Tissue proteomics using chemical immobilization and mass spectrometry. Anal Biochem.

[3] Zhang W, Sakashita S, Taylor P, Tsao MS, Moran MF (2015). Comprehensive proteome analysis of fresh frozen and optimal cutting temperature (OCT) embedded primary non-small cell lung carcinoma by LC-MS/MS. Methods.

[4] Weston LA, Hummon AB (2013). Comparative LC-MS/MS analysis of optimal cutting temperature (OCT) compound removal for the study of mammalian proteomes. Analyst.

[5] Zhao X, Huffman KE, Fujimoto J, Canales JR, Girard L, Nie G (2017). Quantitative proteomic analysis of optimal cutting temperature (OCT) embedded core-needle biopsy of lung cancer. J Am Soc Mass Spectrom.

[6] Bremnes RM, Donnem T, Al-Saad S, Al-Shibli K, Andersen S, Sirera R (2011). The role of tumor stroma in cancer progression and prognosis: emphasis on carcinoma-associated fibroblasts and non-small cell lung cancer. J Thor Oncol.

[7] Ma XJ, Dahiya S, Richardson E, Erlander M, Sgroi DC (2009). Gene expression profiling of the tumor microenvironment during breast cancer progression. Breast Cancer Res..

[8] Place AE, Jin Huh S, Polyak K (2011). The microenvironment in breast cancer progression: biology and implications for treatment. Breast Cancer Res.

[9] Polyak K, Kalluri R (2010). The role of the microenvironment in mammary gland development and cancer. Cold Spring Harbor Perspect Biol..

[10] Ramamonjisoa N, Ackerstaff E (2017). Characterization of the tumor microenvironment and tumor-stroma interaction by non-invasive preclinical imaging. Front Oncol.

[11] Kozlova N, Grossman JE, Iwanicki MP, Muranen T (2020). The interplay of the extracellular matrix and stromal cells as a drug target in stroma-rich cancers. Trends Pharmacol Sci..

[12] Egeblad M, Nakasone ES, Werb Z (2010). Tumors as organs: complex tissues that interface with the entire organism. Dev Cell.

[13] Burden DW (2012). Guide to the disruption of biological samples-2012. Random Primers.

[14] Leica Microsystems. Laser microdissection microscopes: Leica LMD6 & LMD7. https://www.leica-microsystems.com/products/light-microscopes/p/leica-lmd7/. Accessed 15 May 2020.

[15] Golubeva Y, Salcedo R, Mueller C, Liotta LA, Espina V (2013). Laser capture microdissection for protein and NanoString RNA analysis. Methods Mol Biol.

[16] Bevilacqua C, Ducos B (2018). Laser microdissection: A powerful tool for genomics at cell level. Mol Aspects Med.

[17] de Lartigue J (2018). Tumor heterogeneity: a central foe in the war on cancer. J Commun Support Oncol.

[18] Clair G, Piehowski PD, Nicola T, Kitzmiller JA, Huang EL, Zink EM (2016). Spatially-resolved proteomics: rapid quantitative analysis of laser capture microdissected alveolar tissue samples. Sci Rep.

[19] Davis S, Scott C, Ansorge O, Fischer R (2019). Development of a sensitive, scalable method for spatial, cell-type-resolved proteomics of the human brain. J Proteome Res.

[20] Staunton L, Tonry C, Lis R, Finn S, Leary JO, Loda M (2016). Profiling the tumor microenvironment proteome in prostate cancer using laser capture microdissection coupled to LC-MS-A technical report. EuPA Open Proteom.

[21] Nyalwidhe JO, Grzesik WJ, Burch TC, Semeraro ML, Waseem T, Gerling IC (2017). Comparative quantitative proteomic analysis of disease stratified laser captured microdissected human islets identifies proteins and pathways potentially related to type 1 diabetes. PLoS ONE.

[22] Goldhirsch A, Winer EP, Coates AS, Gelber RD, Piccart-Gebhart M, Thürlimann B (2013). Personalizing the treatment of women with early breast cancer: highlights of the St Gallen International Expert Consensus on the Primary Therapy of Early Breast Cancer 2013. Ann Oncol.

[23] Kondov B, Milenkovikj Z, Kondov G, Petrushevska G, Basheska N, Bogdanovska-Todorovska M (2018). Presentation of the molecular subtypes of breast cancer detected by immunohistochemistry in surgically treated patients. Open Access Maced J Med Sci.

[24] Mertins P, Mani DR, Ruggles KV, Gillette MA, Clauser KR, Wang P (2016). Proteogenomics connects somatic mutations to signalling in breast cancer. Nature.

[25] Ritchie ME, Phipson B, Wu D, Hu Y, Law CW, Shi W (2015). limma powers differential expression analyses for RNA-sequencing and microarray studies. Nucleic Acids Res.

[26] Gentleman RC, Carey VJ, Bates DM, Bolstad B, Dettling M, Dudoit S (2004). Bioconductor: open software development for computational biology and bioinformatics. Genome Biol.

[27] da Huang W, Sherman BT, Lempicki RA (2009). Systematic and integrative analysis of large gene lists using DAVID bioinformatics resources. Nat Protoc.

[28] Subramanian A, Tamayo P, Mootha VK, Mukherjee S, Ebert BL, Gillette MA (2005). Gene set enrichment analysis: a knowledge-based approach for interpreting genome-wide expression profiles. Proc Natl Acad Sci USA.

[29] Gene Set Enrichment Analysis. Molecular Signatures Database v6.2. https://www.broadinstitute.org/gsea/msigdb. Accessed 11 July 2019.

[30] Hänzelmann S, Castelo R, Guinney J (2013). GSVA: gene set variation analysis for microarray and RNA-seq data. BMC Bioinform.

[31] Huong PT, Nguyen LT, Nguyen XB, Lee SK, Bach DH (2019). The role of platelets in the tumor-microenvironment and the drug resistance of cancer cells. Cancers.

[32] Bissell MJ, Radisky D (2001). Putting tumours in context. Nat Rev Cancer.

[33] Dudley AC (2012). Tumor endothelial cells. Cold Spring Harbor Perspect Med.

[34] Ericsson C, Franzén B, Nistér M (2006). Frozen tissue biobanks. Tissue handling, cryopreservation, extraction, and use for proteomic analysis. ActaOncol..

[35] Haque R, Ahmed SA, Inzhakova G, Shi J, Avila C, Polikoff J (2012). Impact of breast cancer subtypes and treatment on survival: an analysis spanning two decades. Cancer EpidemiolBiomarkPrev.

[36] McGuire A, Lowery AJ, Kell MR, Kerin MJ, Sweeney KJ (2017). Locoregional recurrence following breast cancer surgery in the trastuzumab era: a systematic review by subtype. Ann Surg Oncol..

[37] Metzger-Filho O, Sun Z, Viale G, Price KN, Crivellari D, Snyder RD (2013). Patterns of recurrence and outcome according to breast cancer subtypes in lymph node-negative disease: results from international breast cancer study group trials VIII and IX. J Clin Oncol..

[38] Voduc KD, Cheang MC, Tyldesley S, Gelmon K, Nielsen TO, Kennecke H (2010). Breast cancer subtypes and the risk of local and regional relapse. J Clin Oncol..

[39] Shriver CD (2010). 21st century paradigm of tissue banking: the Clinical Breast Care Project. Mil Med..

