# Supplementary figures and images for: Comparative analysis of differentially abundant proteins quantified by LC–MS/MS between flash frozen and laser microdissected OCT-embedded breast tumor samples
Source: Clin Proteomics. 2020 Nov 7;17:40. doi: 10.1186/s12014-020-09300-y (PMC7648272; doi:10.1186/s12014-020-09300-y)

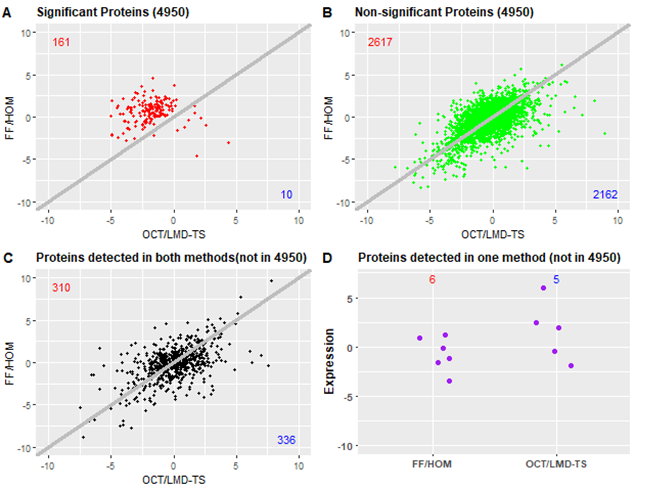

Supplement: Supplementary file 1 — Additional file 1: Figure S1. Scatter plots of normalized protein expression values of sample S2 in FF/HOM vs. OCT/LMD-TS processing methods. Each dot represents one protein. Numbers in red represent the number of more abundant proteins in FF/HOM whereas numbers in blue represent the number of more abundant proteins in OCT/LMD-TS. A, Scatter plot of the reported significant proteins. B, Scatter plot of the non-significant proteins from the 4,950 proteins detected across all samples. C, Scatter plot of the proteins detected in both methods but not in the 4,950 commonly detected proteins. D, Scatter plot of the proteins detected in only one of the methods. [file 12014_2020_9300_MOESM1_ESM.png]
